# Comparison of ring instruments and classic circumcision methods: a systematic review and meta-analysis

**DOI:** 10.1080/2090598X.2022.2071545

**Published:** 2022-05-23

**Authors:** Yavuz Güler, Gökhun Çağdaş Özmerdiven, Akif Erbin

**Affiliations:** a Urology Department, İstanbul Rumeli University, Private Safa Hospital, İstanbul, Turkey; b Urology Department, İstanbul Aydın Üniversitesi, VM Medical Park Hospital, İstanbul, Turkey; c Urology Department, Haseki Training and Research Hospital, İstanbul, Turkey

**Keywords:** Circumcision, ring devices, plastibell, prepex, shang ring

## Abstract

**Aim:**

To determine the advantages and disadvantages of both methods by comparing classic circumcision methods with circumcision methods assisted by ring instruments.

**Material-Methods:**

Only studies that compared open procedures and ring devices for male circumcision were included. A total of 6226 patients were examined in 14 studies. The methodological quality of RCT was evaluated using Cochrane collaboration’s tools. The Review Manager software statistical package was used to analyze the ORs for dichotomous variables and the mean differences for continuous variables. The proportion of heterogeneity across the studies was tested using the I 2 index. Potential publication bias was assessed by identifying the presence of visual asymmetry/symmetry with funnel plot studies.

**Results:**

There were 1812 patients in the open circumcision group and 4414 patients in the ring groups. In total, there was no difference identified between the groups. The open procedure had an advantage compared to the Plastibell subgroup for hemorrhage, while in the other two subgroups, the ring instrument groups had the advantage. Statistically significant in favor of ring devices was found in operating time.There was no difference between the groups for early (postoperative) pain scores. For late-period pain scores, differences with statistical significance were identified in favor of ring devices both in subgroups and in total. For satisfaction, apart from one study in the PrePex group, statistical significance was obtained in favor of ring devices for the other subgroups and in total.

**Conclusion:**

The main factors in favor of the use of ring instruments for circumcision are the short total surgical duration, not requiring advanced surgical experience, ease of learning and application, and patient relative satisfaction rates. However, it is a condition to know open circumcision methods and to have experience of this surgery for use in situations with hemorrhage complications, mainly, and without ring instruments of appropriate size.

## Introduction

Circumcision is the most common surgical procedure performed on male children, and it is predicted that one in every three men globally are circumcised. Circumcision surgery extends back 15,000 years. A variety of studies defined the benefits of circumcision. Penis cancer and cervical cancer risk in partners is reduced. Additionally, it is reported that the risk of catching HIV infection reduces by up to 60% [1–3]. Within the scope of long-term HIV prevention strategies, the World Health Organization (WHO) and the Joint United Nations Program on HIV/AIDS (UNAIDS) recommended adult circumcision along with neonatal circumcision [4].Circumcision is performed in neonatal infants, children and adult males for religious, cultural and medical reasons. It is a radical treatment choice for medical problems like phimosis that cannot be treated by other treatments, balanopostitis and chronic urinary tract infections.

As with every surgical procedure, circumcision has some of its own specific complications. These are minor and treatable complications like hemorrhage, pain, edema and inadequate skin removal commonly observed in the early period. However, serious complications like severe hemorrhage requiring reoperation and amputation of the glans penis may be observed. In the late period, pain, wound site infection, adhesions, meatal stenosis, fistula, loss of penile sensitivity and sexual dysfunction may be observed [5].

Currently, circumcision is performed with a range of methods. Dorsal slit, Gomco clamp, Mogen clamp, bone cutter and Plastibell are the main methods [6]. Due to advantages like being quick, easy to perform, a less traumatic technique with minimal blood loss, lower complication rates and high cosmetic satisfaction, circumcision performed with ring instruments is a very popular and frequently chosen method. While Plastibell ring devices are used in the pediatric age group [7], the PrePex and Shang Ring devices are used in adults over 18 years of age.

In this context, we attempted to perform a systematic review and meta-analysis about the comparative effectiveness of ring device and open procedure circumcisions by collecting all relevant published studies to provide a comprehensive survey that addresses this controversy.

## Materials and methods

### Data sources and search strategy

We searched PUBMED, EMBASE, Cochrane Database of Systematic Review, Web of Science and Google Scholar from their inception until December 2021. These arch terms used to identify potentially eligible studies from each data source were as follows: ‘circumcision’, ‘dorsal slit’, ‘ring devices’, ‘plastibell’, and ‘open circumcision’. The reference lists of the relevant studies were also searched. Two of our authors independently screened all citations and abstracts identified by these arch strategy to screen eligible studies. Only English was used as the language for screening.

### Data extraction, inclusion and exclusion criteria

Only studies that compared open procedures and ring devices with male circumcision status were included. All relevant studies identified from these arch strategy were used for detailed assessment. Case reports, case series, articles not written in English, articles without full text found or accessible and studies comparing circumcision methods apart from the classic open circumcision methods (dorsal slit, sleeve circumcision, forceps-guided) with other ring devices (Mogen, Gomko clamp, guillotine, etc.) were excluded from the study. Data were extracted from the included studies by the authors. The extracted data included data sources, eligibility, methods, participant characteristics, interventions and results.

### Assessment of study quality

The methodological quality of RCT was evaluated using Cochrane collaboration tools, including 6 items: randomization of reviewers, allocation concealment, blinding of personnel and participants, blinding of outcome measurement, incomplete outcomes, selective reporting and other bias [8]. The methodological quality of retrospective and prospective non-randomized studies was evaluated using the modified Newcastle-Ottawa scale(NOS), in which a score of 1–9 stars was allocated [9]. This study followed the PRISMA (preferred reporting items for systematic reviews and meta-analyses) statement [10].

### Data synthesis and analysis

Primary and secondary outcomes were calculated from the estimates of each study to enumerate pooled odds ratios (ORs) and confidence intervals (CIs). The Review Manager 5.3 software (Cochrane Collaboration, Oxford, UK) statistical package was used to analyze the ORs for dichotomous variables and the mean differences for continuous variables. Meta-analyses were performed using this software to determine the ORs and CIs for the following criteria: bleeding, infection, operating time, overall complications, satisfaction, early and late pain scores. The proportion of heterogeneity across the studies was tested using the *I* ^2^ index (range: 0%–100%). If *I ^2^ *< 50%, the variation of the studies was considered to be homogenous and the fixed-effect model was adopted. If *I^2^ *> 50%, the variation of studies was considered to be significantly heterogeneous and the random-effect model was adopted. All P values were two-tailed, and p < 0.05 was considered statistically significant.

### Publication bias

Potential publication bias was assessed by identifying the presence of visual asymmetry/symmetry with funnel plot studies.

## Results

### Search results and study characteristics

**Figure 1.**
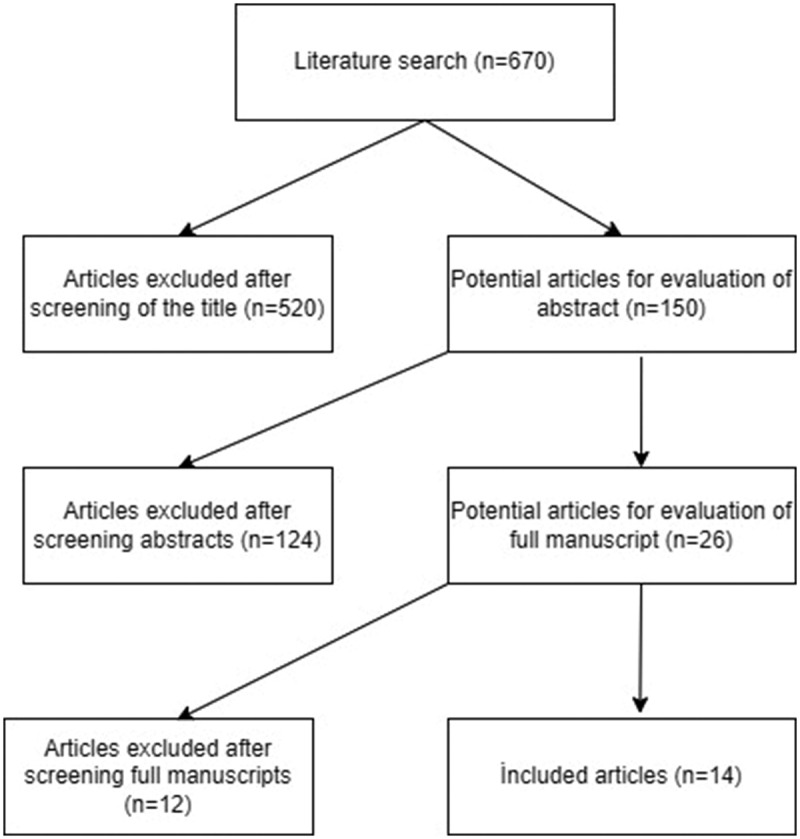
Flow chart of the study.

The details about the literature search and screening process can be found in Figure 1. Upon completion of primary screening by scanning titles and abstracts, the full texts of 14 potentially relevant studies were identified [11–25]. Two were retrospective, 3 were prospective non-randomized and 9 were prospective, randomized and controlled (PRC) studies. The characteristics of the studies are shown in Table 1. The included studies were published between 2008 and 2020. A total of 6226 participants were included from Brazil, Nigeria, India, China, Pakistan, Kenya, Zambia, Uganda, Rwanda and Iran. The number of participants in each study was 60–2441. The follow-up durations for the studies were 5–90 days.

**Table 1.** Demographic and quality data of studies.

Study	Country	Study interval	Study Type	Age, mean(range)		No of patients		Follow up	Quality score (NOS)
				Open	Ring	Open	Ring		
Abdullah, 18	Nigeria	2013	RCT	7d-10y	7d-10y	60	60	N/A	−
Modı, 21	İndia	2017–2019	Retro	4.77 ± 2.4(y)	4.8 ± 2.2(y)	30	30	N/A	5 star
Gavade, 20	İndia	2017–2019	RCT	3.1 y <1: 26% 1–5: 53% 6–10: 17% 11–15: 3%	3.7 y 35% 48% 17% 0%	58	58	N/A	−
Hamza, 20	Nigeria	2016–2017	RCT	3 m (8d-5y)	1 m (9d-5y)	55	55	1 m	−
Talini, 18	Brasil	2015–2016	Retro	5.27 (10 m-13 y)		501	1940	N/A	5 star
Mouniddin,18	İndia	2016–2018	RCT	3.5 ± 2.8 (m)	4.0 ± 3.5 (m)	250	310	15th day (open group), on day of separation of the ring (ring group)	−
Lei, 16	China	2012–2014	Pros NR	18–44:(69.7%) 45–59: (25%) 60–76:5.3%	75% 20% 5%	76	306	4 w	5 star
Mahmood, 15	Pakistan	2012–2014	RCT	1 w-2y	1 w-2y	50	50	5 d	−
Sokal, 14	Kenya, Zambia	2011	RCT	20.5(18–39) (m)	21(18–41) (m)	201	197	60 d	−
Kigozi, 14	Uganda	2012–2013	Pros NR	18–25 (m): 65% 25–29 (m): 17% 30–34 (m): 8% 35–39 (m): 4% 40+ (m): 8%	67% 15% 8% 3% 8%	68	326	4 w (open group) 7 w (ring group)	5 star
Kigozi, 13	Uganda	N/A	Pros NR	<24 (m): 68% 25–29 (m): 14% 30–34 (m): 10% 35–39 (m): 7% >40 (m): 2%	63% 16% 7% 8% 7%	117	500	4 w	5 star
Mutabazi, 12	Rwanda	2011	RCT	24 ± 4.1(21–54) (m)	26 ± 5.2(21–54) (m)	73	144	9 w	−
Netto, 10	Brasil	2006–2008	RCT	72 ± 32 (m)	71 ± 32 (m)	68	57	87(64–102) d	−
Mousavi, 08	Iran	2002–2008	RCT	4.4 ± 3.2 (m)	3.1 ± 2.8 (m)	205	381	N/A	−

## Assessment of study quality

**Figure 2.**
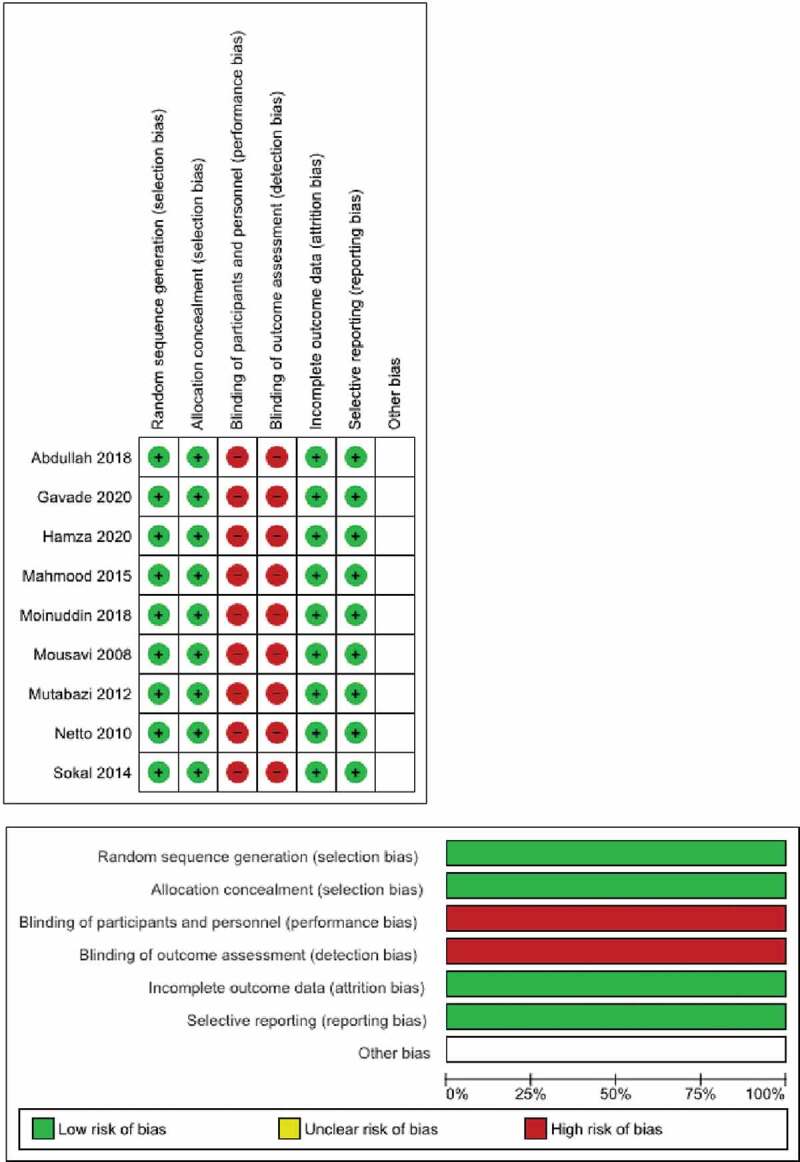
Cochrane collaboration tools study chart.

Quality assessment was performed according to NOS for 2 retrospective and 3 prospective non-randomized studies. The 5 studies were assessed as having fair quality with 5 stars each. Prospective randomized controlled studies were rated as high risk for questions about blinding of participants and personnel (performance bias) and blinding of outcome assessment (detection bias) as surgical methods preclude blinding(Figure 2).

## Primary outcomes

In studies comparing Plastibell ring device and open procedures, mean age extended from the neonatal period to 15 years of age. In studies comparing the Shang Ring [11,13], PrePex device [12,14] and open procedures, the studies included adult males over 18 years. Comparisons with open procedures used the Plastibell in 9 studies, PrePex in 2 studies and Shang Ring in 3 studies.

### Operating times

**Figure 3.**
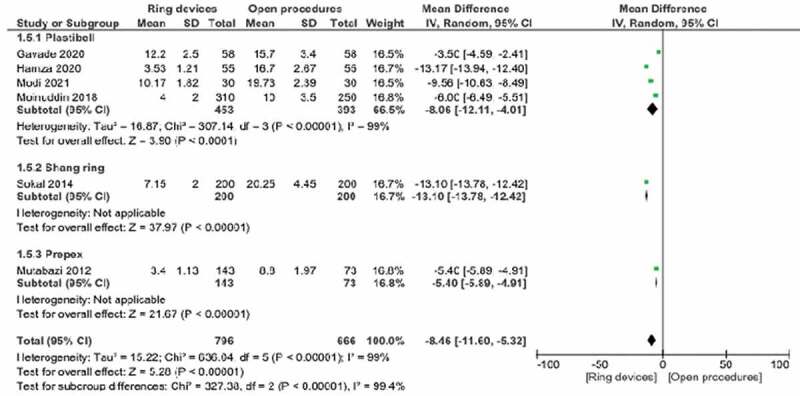
Forest plot for operation time - Weighted odds ratio 95% CI -8.46(-11.6,-5.32).

This parameter was included in 9 studies. Statistical significance was present in favor of ring devices (p < 0.01). Weighted odds ratio 95% CI 1.45 (0.51, 2.58) (Figure 3,Table 2).

**Table 2.** Operative and postoperative data.

Open/Ring devices															
Studies	Operation time (min)	Total complications(%)	İnfection(%)	Bleeding(%)	Edema (no)	Adhesion (%)	Cicatricical (%)	Wound dehiscence (no)	Wound healing time(day)	Excess mucosa (no)	Pain scores early	Pain scores late	Satisfaction(%)	Procedures performed by	Anesthesia type
Abdullah,18	12/7	N/A	1.7/2.6	1.7/5	N/A	N/A	N/A	N/A	8/10	N/A	N/A	N/A	91	Surgeon	N/A
Modi,21	19.73 ± 2.3/10.17 ± 1.82	N/A	3.3/3.3	0/3.3	N/A	N/A	N/A	N/A	N/A	0/1	N/A	N/A	N/A	Trained resident doctors	Local/General
Gavade,20	N/A	N/A	N/A	1.7/5.2	N/A	N/A	N/A	N/A	N/A	N/A	N/A	N/A	N/A	Surgeon	N/A
Hamza,20	N/A	9.1/29.1	N/A	0/9.1	N/A	1/0	N/A	N/A	N/A	N/A	N/A	N/A	N/A	Surgeon	Local: < 1 year age General: > 1 year age
Talini,18	N/A	3/3.4	N/A	0.6/1.2	N/A	N/A	N/A	N/A	N/A	N/A	N/A	N/A	N/A	Surgeon	General
Monuiddin,18	10 ± 3.5/4 ± 2	8/21	3.2/5.2	4/8.1	N/A	N/A	N/A	N/A	N/A	2/4	N/A	N/A	N/A	Surgeon	Local
Lei,16	23.4 ± 4.3/4.8 ± 0.9	N/A	9.2/2.9	13.2/1	3/29	N/A	N/A	0/3	N/A	N/A	3.1 ± 1.4/1.8 ± 1.3	5.8 ± 1.4/4.0 ± 1.2	72.3/96.4	Surgeon	Local
Mahmood,15	N/A	N/A	4/0	6/0	N/A	N/A	N/A	N/A	N/A	N/A	N/A	N/A	N/A	Surgeon	Local
Sokal,14	29.5 ± 4.5/7.2 ± 2.0	N/A	1/0	0.5/0	0/1	N/A	N/A	4/6	38.6 ± 12.6/44.1 ± 12.6	N/A	N/A	N/A	78.6/96.3	Non-physicians 82% Phsicians and nonphysician 17% Physicians 2%	Local
Kigozi,14	N/A	0/9	N/A	N/A	0/1	N/A	N/A	N/A	98.7%(at 4 week)/98.6%(at 7 week)	N/A	N/A	N/A	N/A	Clinical officers	Local gel (For prepex group)
Kigozi,13	17.7 ± 7.3/6.1 ± 2.7	3/14	0.85/0	0/0.2	0/1	N/A	N/A	1/1	N/A	0/1	N/A	N/A	100/99.1	Clinical officers	Local
Mutabazi,12	8.8 ± 2.0/3.4 ± 1.1	11/2.7	N/A	19.2/0.7	11/21	N/A	N/A	N/A	23 ± 7.5/31.0 ± 12.1	N/A	3.8 ± 2.1/5.6 ± 1.8	5.2 ± 2.2/2.5 ± 1.8	N/A	Surgeon	Local
Netto,10	14.6 ± 1.9/3.3 ± 1.5	26/10.5	0/0	10.3/5.3	N/A	29.4/10.5	8.8/3.5	N/A	N/A	N/A	Use of paracetamol was similar in the firsth 2 days.	Plastibell group reguired more painkiller	N/A	Surgeon	General
Mousavi,08	N/A	N/A	0/1	2/1.3	N/A	N/A	N/A	N/A	N/A	0/5	N/A	N/A	N/A	Surgeon	Local

### Overall complications

**Figure 4.**
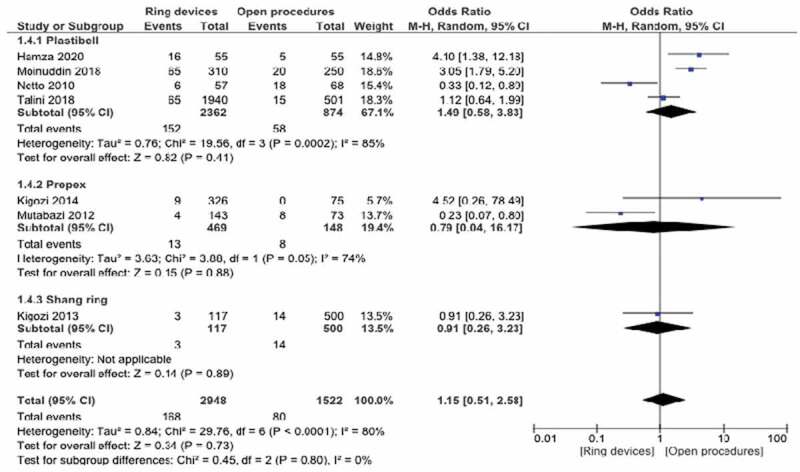
Forest plot for overall complications - Weighted odds ratio 95% CI 1.15(0.51,2.58.

Overall complications were considered in a total of 7 studies, 4 with Plastibell, 2 with PrePex and 1 with Shang Ring. Statistical significance was not present for subgroups or in total on the meta-analysis (p = 0.69). Weight odds ratio 95% CI 1.18 (053–2.63) (Figure 4,Table 2).

### Satisfaction

**Figure 5.**
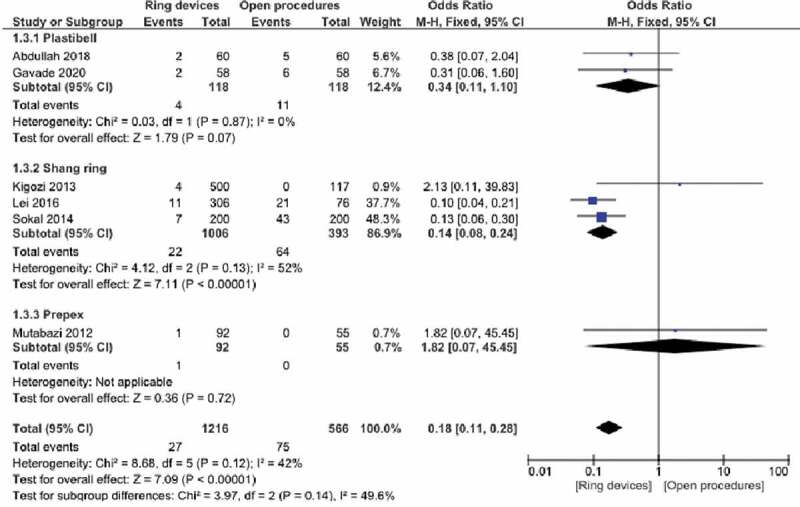
Forest plot for satisfaction.

Satisfaction scoring was performed in 6 studies. In terms of subgroups, there were 2 studies using Plastibell, 3 studies using Shang Ring and 1 study using PrePex. Apart from the single study in the PrePex group, statistical significance was obtained in favor of ring devices in the other subgroups and in total (P < 0.01). Weighted odds ratio 95% CI 0.18 (0.11, 0.28) (Figure 5,Table 2).

### Bleeding

**Figure 6.**
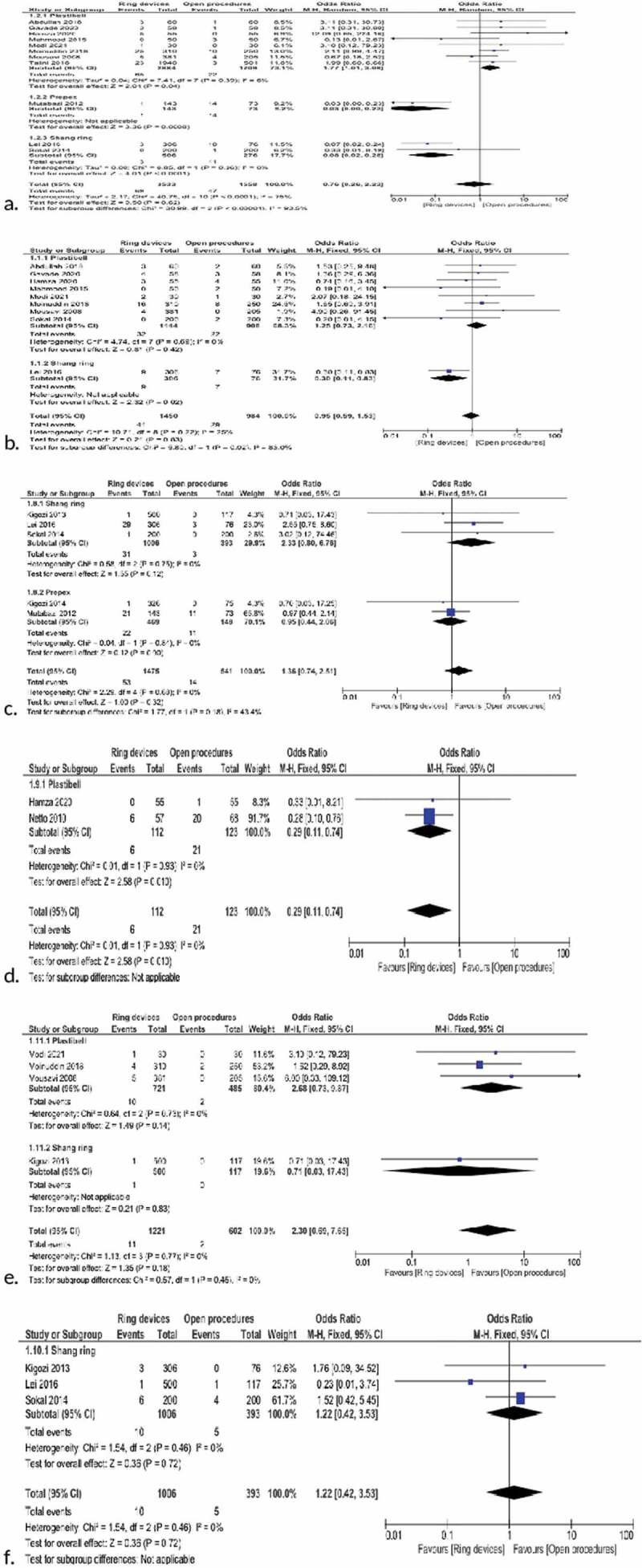
Forest plot for a. Bleeding, b. Ä°nfection, c. Edema, d. Adhesion, e. Ä°nsufficient skin removal, f. Wound dehiscence.

This parameter was recorded in a total of 13 studies (Table 2). Data related to hemorrhage complications were found in 8 studies using Plastibell, 1 study using PrePex and 3 studies using Shang Ring. While there was an advantage in favor of the open procedure for the Plastibell subgroup (p = 0.04), the advantage was in favor of the ring devices for the other two subgroups (p < 0.001). In total, there were no differences identified between the groups (P = 0.59). Total weighted odds ratio 95% CI 0.75 (0.27–2.10) (Figure 6a,Table 2).

### Infection

This was reported in a total of 9 studies, with 8 using Plastibell and 2 using Shang Ring. There was no statistical significance for the open vs. ring group for the Plastibell subgroup (P = 0.42). For the Shang Ring group, the difference was in favor of the ring device (P = 0.005). No statistical difference was present in total (P = 0.64). Weighted odds ratio 95% CI 0.89 (0.56–1.43) (Figure 6b,Table 2).

### Edema

This was investigated in 3 studies from the Shang Ring group and 2 studies from the PrePex group. Edema reports were not encountered in any study from the Plastibell group. Statistically significant differences were not identified for the subgroups or in total (P = 0.32). Weighted odds ratio 95% CI 1.36 (0.74,2.51) (Figure 6c,Table 2).

### Adhesion

In the subgroups, only 2 studies from the Plastibell group reported adhesion. There was a statistically significant difference in favor of the Plastibell group for comparisons between Plastibell devices and open procedures (P = 0.01). Weighted odds ratio 95% CI 0.29 (0.11,0.74) (Figure 6d,Table 2).

### Insufficient skin removal

Inadequate tissue removal was reported in 3 studies in the Plastibell subgroup and 1 study in the Shang Ring subgroup. Statistical differences were not present between open procedures and ring devices in the subgroups or in total (P = 0.18). Weighted odds ratio 95% CI 2.3(0.39,7.65)(Figure 6e,Table 2).

### Wound dehiscence

Data related to wound dehiscence were only accessed in three studies in the Shang Ring group. Significant statistical differences were not identified between the open procedure and Shang Ring group (p = 0.72). Weighted odds ratio 95% CI 1.22 (0.42, 3.53) (Figure 6f,Table 2).

### Early (perioperative) pain scores

**Figure 7.**
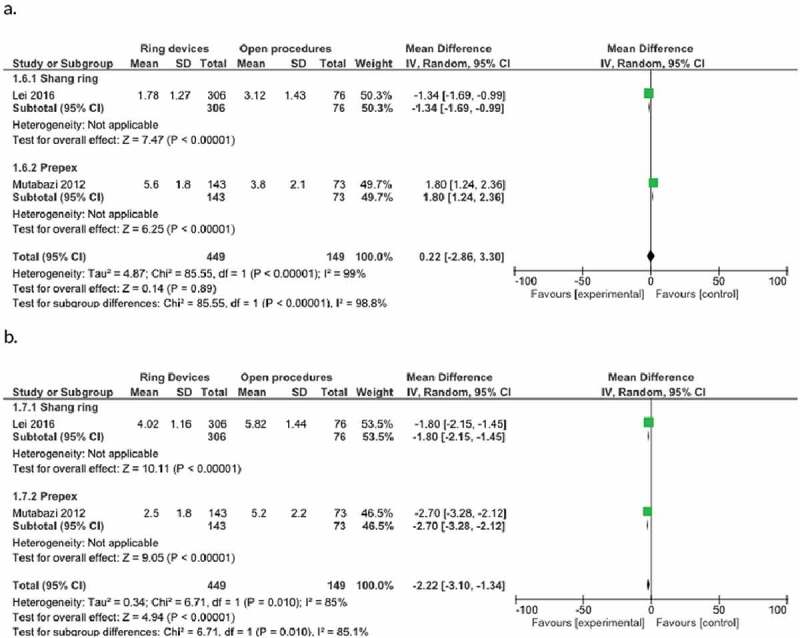
Forest plot for painscores. a. Early, b. Late.

Only one study in the Shang Ring and one study in the PrePex subgroup assessed pain scoring in the early postoperative period. While differences were identified between the subgroups, a statistically significant difference was not identified in total (p = 0.89). Weighted mean difference 0.22, 95% CI −2.86 to 3.30 (Figure 7a,Table 2).

### Late pain scores

Only one study in the Shang Ring and one study in the PrePex subgroup performed pain scoring studies in the late postoperative period. Statistical significance in favor of ring devices was identified in both subgroups and in total (p < 0.01). Weighted mean difference −2.22, 95% CI −3.10 to −1.34 (Figure 7b,Table 2).

## Outcomes related to ring instruments

Spontaneous ring removal time was given in a total of 5 studies [13,17,19,22,24]. In the Plastibell group, 3 studies [19,22,24] had similar spontaneous separation durations (5.2, 6 and 6.2 days, respectively). Only one study reported 16 days [17]. The fifth study was in the Shang Ring group and the mean separation duration was 18 days [13]. The spontaneous separation rate was given in 8 studies [16,17,19–22,24]-Plastibell; 13-Shang Ring Group). The lowest spontaneous separation rate in the Plastibell group was 70% [20], while another study had rate of 85.5% [22] and the other studies were all above 90%.The other study in the Shang Ring group had spontaneous separation rate of nearly 80%.

In 2 studies, data about ‘device removal by participants’ were given [11,15]. Ring devices were reported to be removed by participants at rates of 0.6% in the Shang Ring study [11] and 1.7% in the PrePex study [15]. Two studies considered whether there was a correlation between age and ring separation rates [16,22]. In both studies, as age increased, spontaneous separation was reported to be delayed. Only one study assessed the correlation between ring diameter and separation duration and found no correlation [22]. The same study examined the correlation between age and complications and reported that as age increased, complication rates increased. Another study researching the correlation between patient body weight and spontaneous ring separation reported that as weight increased, separation was delayed [16] (Table 3).

**Table 3.** Ring instrument specific data.

Study	Ring Device Type	Spontaneous Ring Separation Rate (%)	Spontaneous(sp) Ring Removing Time	Displacement or Migration of Ring Devices(%)	Correlation’s	Device removed by participant (%)
Modi 2021	Plastibell	93.4	0–5 years: 4.4 ± 1.0 6–10 years: 8.0 ± 1.6 Total: 5.2 ± 1.9 (day)	3.3		N/A
Hamza 2020	Plastibell	85.5	Neonates: 5.7 ± 2.0 (spontan/sp) 1–11 month:7.1 ± 2.6(sp) 1–4 years:10.5 ± 0.7(sp) >5: 14 day (Surgical) Total: 6(2–11)	N/A	-Correlation between the size of the ring and number of days for separation: No -Correlation between age of subjects and bell separation time: Yes -Correlation between age and freguency of complications:Yes	N/A
Gavade 2020	Plastibell	N/A	N/A	N/A		N/A
Abdullah 2018	Plastibell	98.2	N/A	1.7		N/A
Talini 2018	Plastibell	70	N/A	1.7		N/A
Mouniddin 2018	Plastibell	96.5	6.2(3–12)(day)	2.9		N/A
Mahmood 2015	Plastibell	N/A	N/A	N/A		N/A
Netto 2010	Plastibell	100	16 ± 5(6–30)(day)	0		0
Mousavi 2008	Plastibell	97.4	N/A	0.5	Correlation between weight of subjects and bell separation time:Yes -Correlation between age of subjects and bell separation time: Yes	N/A
Lei 2016	Shang	79.2	18 ± 6 (day)	0.7		N/A
Sokal 2014	Shang	0	Surgical	0		0
Kigozi 2013	Shang	0	Surgical	0.8		0.6
Kigozi 2014	Prepex	0	Surgical	0.3		1.7
Mutabazi 2012	Prepex	0	Surgical	0		0

## Other outcomes

**Figure 8.**
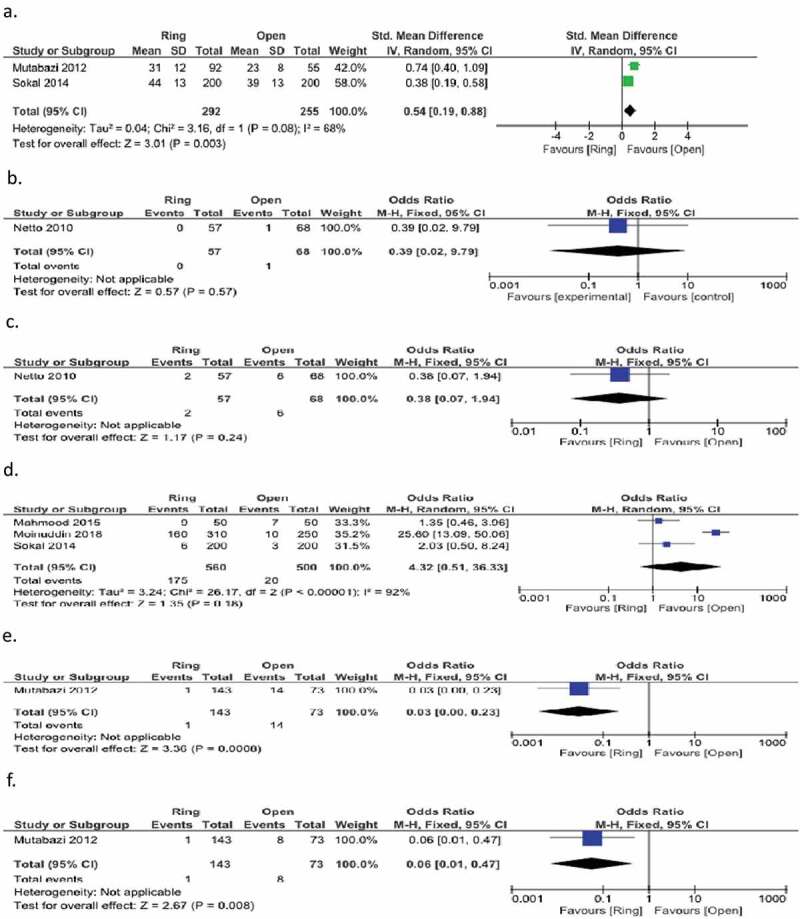
Forest plot for a. Complet wound healing, b. Reoperation, c. Cicatrical, d. Postprocedural pain, e. Oozing, f. Clear exudate.

Two studies considered complete wound healing. These studies revealed a statistical advantage for the classic circumcision group compared to the ring group (SMD IVR 95% CI; 0.54(0.19,0.88), p = 0.003) [12,14] (Figure 8a). Kigozi et al. [15] reported wound healing rates in the 4th and 7th weeks. While 98.7% full healing was observed in the open group in the 4th week, this rate was 56.7% in the ring group. By the 7th week, the healing rate in the ring group had reached 98.6%.

Only one study gave reoperation and cicatricial data [17]. In the ring and open circumcision groups, there was no statistically significant difference for both parameters (reoperation and scar p values 0.57 and 0.24, respectively) (Figure 8b,c). Three studies considered postprocedural pain rates [12,18,19]. In terms of postprocedural pain rates, there was no statistically significant difference found between the groups (p = 0.18) (Figure 8d). One study considered oozing and clear exudate [14]. The ring group was found to be statistically advantaged for both parameters, (p values 0.0008 and 0.008, respectively) (Figure 8e,f).

## Discussion

Circumcision is performed for medical (phimosis, paraphimosis, balanopostitis, etc.), cultural and religious reasons in the world in general [25]. Geographies where circumcision is performed generally have dense populations and are countries with low income structure socioeconomically. For these reasons, it is important to be able to perform the circumcision procedure more rapidly with low cost. Due to these features, ring devices have gained popularity for circumcision [26,27]. Plastibell ring devices are used with internal diameter 1–1.7 cm for the neonatal-infant and pediatric age group. In recent times, ring devices have begun to be used for adult circumcisions. Among these, the main examples are PrePex and Shang Ring devices.

It appears the most important advantage of ring devices is the short duration of the procedure for circumcision [28]. This situation may reduce stress for patients undergoing circumcision with local anesthesia, especially. Additionally, it may ensure reductions in extra costs like anesthetic drug amounts and laryngeal mask used during circumcisions performed under general anesthesia. In this meta-analysis, the operation duration for studies in the ring group was 3.3–10.2 min, while the duration for the open procedure group was 10–23.4 min (p < 0.001). Classic circumcision is a complex surgery including cutting of the prepuce skin, bleeding control and primary suturing of skin-mucosa and requires serious experience and long procedure times [29]. Additionally, considering that most circumcision procedures in the world in general are performed under local anesthesia (without much comfort), the need for a circumcision method with the shortest duration that provides the best outcome is clear. The most serious time advantage of circumcisions with ring devices is that bleeding control and skin-mucosa suturing procedures are not performed [30].

From the studies included, it is understood there was a correlation between circumcision age and Plastibell ring separation duration [16]. Studies by Hamza and Modi observed that as age reduced the ring separation time symmetrically reduced in subgroup investigations according to the ages of circumcised children [22,24]. In similar studies using the Plastibell ring device, as the age of circumcision fell, the ring separation time was reported to decrease [29]. We think this correlation may be due to the prepuce being thinner and easier sloughing. When neonatal, infant and child circumcision are considered for the Plastibell subgroup, mean spontaneous ring separation duration was less than 10 days. This duration was laterin only 1 study, even though the mean age was not different to the other studies [17]. It appears that some authors in the Plastibell subgroup determined a cut-off duration for waiting according to themselves. Abdullah et al. determined the cut-off duration as 12 days, while Hamza et al. determined the cut-off as 14 days [21,22]. At the end of this duration, Hamza observed ring devices had still not separated in 15% of patients, while Abdullah found this was the case for 1.8% of patients. The ring devices that did not separate by the determined day were surgically removed. Nearly all of the rings with delayed separation were in the advanced pediatric age group. Though it appears like a second surgery, most procedures were easily performed with administration of a local anesthetic spray and did not take a long duration. In studies using the Shang Ring and PrePex devices (apart from Lei et al.), the authors apparently did not wait for spontaneous separation of the ring and performed surgical removal due to the reduced probability of spontaneous separation at advanced ages [11–15].

Though observed less in children in the neonatal and infant period compared to older children, complications are observed with all circumcision methods [31]. More complications are reported for circumcision performed by traditional non-medical circumcision providers, especially [31]. Hemorrhage is most commonly observed among early complications. From the advancing postoperative days, complications like infection, adhesion, prepuce stenosis, hypertrophic skin scar, skin separation, and meatal stenosis may be observed, while there may be major complications like glans necrosis and urethra-cutaneous fistula. In addition to these complications specific to the surgical procedure, there appear to be additional complications specific to the device in the ring group (migration, late separation or semi-separation, etc.) [31]. In studies in this meta-analysis, we did not observe a difference in terms of overall complications between the 3 ring devices and the open procedures (P = 0.73).

Hemorrhage is the most common complication observed after circumcision surgery, in spite of the reduction as the age of circumcision falls [30–32]. When examined in total, a difference was not identified between the classic and ring circumcision groups in terms of hemorrhage (p = 0.0.62). However, in subgroup analysis, the classic operations appeared to be more advantageous in terms of hemorrhage compared to the Plastibell ring group (P = 0.04). Contrary to this, circumcision with ring devices appeared more advantageous in the PrePex and Shang Ring subgroups (P < 0.01); however, the low number of studies for comparison in these two subgroups should not be forgotten as it may be misleading. Most postoperative circumcision hemorrhage stops with compression bandaging. However, though rarely, massive hemorrhage requiring re-operation (suture and/or cauterization) may be observed. Probably these hemorrhages are observed due to loosening of sutures, lack of full placement of the internal ring, depth of the dorsal slit incision being below the ring suture and most importantly tearing of the frenula fold by the ring [33]. Choosing a ring of appropriate size for the penis glans diameter and creating sturdy sutures will prevent these hemorrhages. In studies that prevented tearing of the frenulum by changing the ventral portion of the ring device with some modifications, hemorrhage rates were shown to be lower [30]. For this reason, as experience performing circumcision with ring devices increases, we believe the hemorrhage complication rates with reduce further. In open circumcision operations, all open vein ends extending under the skin and above the dartos fascia should be cauterized with bipolar cautery or tiedbefore skin-mucosa primary suturing. Additionally, it is important to cauterize or suture actively weeping sites along the skin incision edges. Sometimes hemorrhage does not stop in spite of compression bandaging [34]. When diluted adrenalin ring block anesthesia is performed, these veins may not bleed due to vascular spasm and it should not be forgotten that they may be overlooked for this reason.

Surficial skin infections may be observed after circumcision. In this meta-analysis, both surgical methods were similar in terms of infection (p = 0.64). When studies with different ring device durations are examined, though we did not identify a difference in rates of patients with infection, we think leaving the ring devices for long durations may increase the risk of infections. Contrary to this, fewer infections were observed in neonates and infants compared to older children [7]. Suboptimal local wound checks at home, activity and inability to restrict contamination among older children may explain the higher observation of infection in this age group. Full adherence to surgical sterility rules by the person performing circumcision is important. Infection rates after circumcisions performed by traditional circumcision providers are known to be higher than for medical practitioners [35]. However, there is benefit in underlining the heterogeneity in infection definitions between studies. Many authors defined infection as clinical infection only, without examining any culture tests from skin swabs. At the same time, presence of pus was not noted in the definition of infection. Authors using prophylactic topical antibiotic ointment applications in the postoperative period explained the very low infection rates [16].

While there was no statistical difference between the groups in terms of postoperative early period pain scores, late period pain scores were statistically significantly higher in the open circumcision group. Studies giving pain score data were observed to be studies in the adult age group. Some patients in the ring group reported describing pain only during erection [13,20]. In the adult group, it was reported the Shang Ring was more advantageous compared to Prepex due to its elastic properties and it could be applied with only local anesthetic sprays without requiring ring or penile block anesthesia [15,20].

Questioning about parental satisfaction found the ring device groups were significantly more advantageous. Families attach great importance to the cosmetic appearance of the penis when healing is complete after circumcision. Factors related more to classic procedures like obvious suture sites on the skin, surrounding edema, asymmetric skin removal, hypertrophic scar tissue and keloids cause the skin of the penis to appear flawed [36]. Regular and symmetric skin removal is possible with ring devices. Falcao et al [37]. assessed the conventional technique with subcuticular stitches (SC) and the Plastibell (PB) groups in terms of healing and aesthetics on the postoperative 30th and 60th days in prospective and randomized studies. Scores were given separately for each patient by a dermatologist, pediatrician and plastic surgeon. The pediatrician and plastic surgeons found the PB group was the group with best healing, while results were similar in aesthetic terms to the SC group.

Adhesions may be observed between the penis skin and mucosa or glans penis after circumcision. Though it appears to be a minor complication, skin adhesions cause a dead cavity for accumulation of smegma and debris. If this is not corrected, it may form an area where infective agents can lodge. However, though rarely, sharp dissection of these skin bridges may be required; generally they may beeasily opened with steroid creams and/or blunt manipulation with the hands. This problem is encountered more frequently in those with buried penis especially and infants using diapers [38]. In this meta-analysis, we noticed that most studies did not report adhesion incidence; the few studies that did report it observed more adhesion with the classic methods [17,22]. However, it is difficult to assess whether the ring groups or classic methods are more advantageous in terms of adhesion. To prevent adhesion, there is benefit in recommending regular manual manipulation by families to prevent skin adhesion especially in infants with buried penis and using diapers. Additionally, it is necessary to check circumcision patients after full wound healing.

When the total healing durations are examined, the ring groups generally emerged as disadvantaged compared to the open group. Probably, the resolution of edema and inflammation occurring due to vascular and lymphatic obstruction caused by the ring device takes longer compared to the open procedure [16,32].

None of the studies included in our meta-analysis reported urinary retention after circumcision with ring devices. Urine retention may be observed due to reasons such as glandular prolapse, excessive stretching of the prepuce and not selecting a ring device with diameter appropriate for the glans [30].

Factors like not studying the cost of operations, circumcisions not being performed only by surgeons, inclusion of 2 retrospective studies and lack of double-blinding of prospective randomized studies may reduce the power of this meta-analysis. However, double-blinding is not possible for surgical procedures like circumcision. Additionally, some prospective studies left the choice of circumcision method to the patient and parents. However, the fact that 9 out of 14 studies were prospective and randomized contributes to the power of this meta-analysis.

In conclusion, though circumcisions with ring devices do not appear to have an advantage in terms of postoperative complications, the most important advantages are the short operation duration, high family satisfaction in terms of cosmetic appearance and ability to be easily learned and performed by assisting health personnel in countries without adequate numbers of professional health employees. However, it is a condition to know open circumcision methods and to have experience of this surgery for use in situations with hemorrhage complications, mainly, and without ring instruments of appropriate size.
